# Poly (2-hydroxyethylmethacrylate –*co*–methylmethacrylate)/Lignocaine Contact Lens Preparation, Characterization, and in vitro Release Dynamic

**DOI:** 10.3390/polym11050917

**Published:** 2019-05-23

**Authors:** Taieb Aouak, Wassem Sharaf Saeed, Nawaf M. Al-Hafi, Abdel-Basit Al-Odayni, Abdulaziz Ali Alghamdi, Idriss Bedja

**Affiliations:** 1 Chemistry Department, College of Science, King Saud University, Box 2455, Riyadh 11451, Saudi Arabia; aalghamdia@ksu.edu.sa (A.A.A); aalodayni@ksu.edu.sa (A.-B.A.-O.); 2 Cornea Research Chair, Department of Optometry, College of Applied Medical Sciences, King Saud University, Riyadh 11433, Saudi Arabia; nawafalhafi@gmail.com (N.M.A.-H.); bedja@ksu.edu.sa (I.B.)

**Keywords:** Poly (2-hydroxyethylmethacrylate-co-methylmethacrylate)/lignocaine drug-carrier system, contact lens, water content, cell viability, mechanical properties, transparency

## Abstract

2-hydroxyethyl methacrylate, methylmethacrylate, ethylene glycol dimethyl methacrylate, and lignocaine (drug) were mixed together and the monomers were copolymerized at 60 °C through a free radical polymerization in the presence of α,α′-Azoisobutyronitrile in tetrahydrofuran. A series of copolymer/drug composites with different monoacrylate monomer compositions were prepared by solvent evaporation and characterized by different methods such as nuclear magnetic resonance, differential scanning calorimetry, Fourier transform infrared, X-ray diffraction, and mechanical and optical testing. The water content in the copolymers and the cell viability test on the samples were also examined in this investigation. The results of the analyses of the properties of this drug-carrier system are promising, indicating that this material may be a potential candidate for contact lens applications. The release dynamic of this medication from the prepared drug-carrier systems was investigated in neutral pH media. The results obtained revealed that the diffusion of lignocaine through the copolymer matrix obeys the Fick model and the dynamic release can be easily controlled by the methyl methacrylate content in the copolymer.

## 1. Introduction

Contact lenses are a commonly used device that corrects refraction and protects the ocular surface. Recently, there has been increased interest in delivering drugs through contact lenses, because common eye drops are inefficient in delivering drugs that penetrate the cornea and thus reach the ocular tissue [1,2]; only 5% of the applied drug penetrates the cornea after eye drop use. Unfortunately, the rest of the drug is not only lost but reaches other organs in the body, specifically the conjunctiva and lacrimal sac, which creates side effects [2,3]. In the worst incidences, the remaining drug reaches the bloodstream through the lacrimal sac and nasolacrimal duct [4]. This shows that the use of drops as a drug delivery system limits the efficacy of the therapeutic system and wide-angle glaucoma treatments, such as the beta-blocker Timolol, have a deleterious effect on the heart [5].

Soaking contact lenses in a drug solution, which loads the drug into the lens, has been used in previous and current attempts for ophthalmic drug delivery; this delivery process usually lasts for one to several hours [6,7,8]. Unfortunately, the drug is released from the contact lens in a short period of time. Therefore, expanded time-release cannot be achieved by this method. In addition, the equilibrium solubility of the drug limits the amount of drug (mostly hydrophobic) that can be loaded into the lens matrix by the soaking process.

Gulsen et al. [9] attempted to develop disposable soft contact lenses as a new vehicle for ophthalmic drug delivery. This group added drug-laden nanoparticles into the matrix of soft poly 2-hydroxyethyl methacrylate (*p*-HEMA) hydrogel lenses. Microemulsions of hexadecane in water around the particles were used to produce transparent hydrogels, and the polymer matrix was synthesized by solution free-radical polymerization in the presence of ethylene glycol-dimethacrylate (EGDMA) [9]. This material, of which the contact lens is made, helps control the release of the drug through the tear film trapped between the cornea and the contact lens. This directional and targeted drug release helps decrease the absorption of the drug in the blood stream through both the lacrimal sac and the nasolacrimal duct. Additionally, the compound took a few days to reach the therapeutic release level. Thus, when using a soft contact lens as a drug-carrier instead of the usual drops with 2–5 min residence time (topical application of drugs), the drug molecules have a much longer residence time in the post lens tear [10,11].

**Scheme 1:**
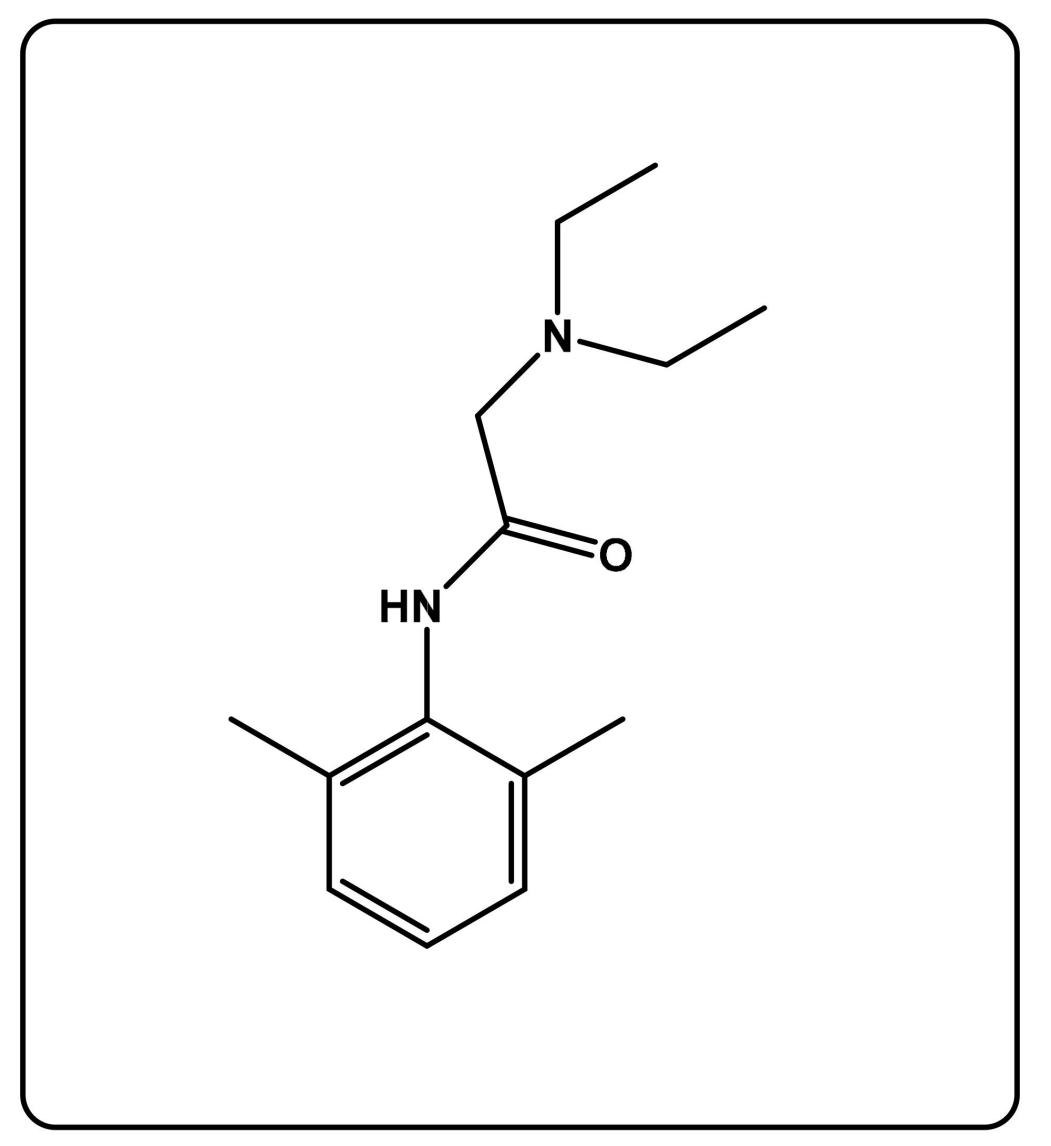
Chemical structure of lignocaine.

In this study, we contribute a new controllable vehicle for ophthalmic drug delivery, in which the dynamic release and the amount of the medication can be easily controlled over time through an adequate hydrophilic/hydrophobic composition of a copolymer. To reach this goal, 2- (diethylamino)-*N*-(2,6-dimethylphenyl) acetamide (lignocaine; LIG) (Scheme 1), which was the chosen drug, was incorporated in poly(2-hydroxyethyl methacrylate-co-methyl methacrylate) crosslinked (PHEMMAC) as a composite, by mixing in situ 2-hydroxyethyl methacrylate (HEMA), methylmethacrylate (MMA), ethylene glycol-dimethacrylate (EGDMA) as crosslinker, and medication together before copolymerization in tetrahydrofuran (THF), in the presence of Azobisisobutrylonitrile (AIBN) as an initiator. A series of LIG/PHEMMAC drug-carrier systems with different monomer compositions were prepared by solvent evaporation and characterized by nuclear magnetic resonance, differential scanning calorimetry, Fourier transform infrared (FTIR), X-ray diffraction, mechanical testing, and UV-visible techniques. The water content in the copolymers and the cell viability test on the samples were also examined in this investigation. The release dynamic of this medication from the prepared drug-carrier systems was investigated in neutral pH media.

## 2. Materials and Methods

### 2.1. Materials

HEMA (purity, 99%), MMA (purity, 98%), EGDMA, AIBN, and THF (purity, 98%) were purchased from Sigma Aldrich (Taufkirchen, Germany). Monomers and crosslinkers were purified from hydroquinone by distillation under reduced pressure and then stored in nitrogen gas atmosphere before use. AIBN, which was used as an initiator, was purified by recrystallization in absolute ethanol. LIG was purchased from the pharmacy as an aqueous solution for eye injection drops (lignocaine hydrochloride injection BP 2% *w*/*v*). The pure LIG drug was extracted by evaporating water at 40 °C under reduced pressure. 1, 1-Diphenyl-2-picryl-hydrazyl (DPPH), Dulbecco’s modified Eagle’s medium (DMEM), fetal bovine serum (FBS), and ascorbic acid were all provided by Sigma Aldrich.

### 2.2. Synthesis of PHEMMA

Poly(2-hydroxyethylmethacrylate-co-methylmethacrylate) (PHEMMA) used as reference in this investigation was synthesized by the free radical copolymerization of HEMA with MMA in THF using AIBN as an initiator. HEMA and MMA were distillated at reduced pressure, degassed, and stored in nitrogen. A defined ratio of the HEMA/MMA mixture in THF was added to 0.1 wt % of AIBN and then mixed together into a two-neck round-bottom flask equipped with a magnetic stirrer, and connected to a reflux condenser and a pipe of dry nitrogen gas. The obtained mixture was then bubbled with dry nitrogen and maintained at 60 °C for 3 h. The obtained viscous solution containing the PHEMMA copolymer was casted overnight onto a Teflon plate and allowed to dry for 24 h at room temperature, after which the entire residual solvent and monomers evaporated at 60 °C under vacuum conditions. The dried films obtained were monitored throughout for stability of their weights, then kept in the shade for further experiments and characterization. A series of PHEMMA copolymers of different compositions were synthesized by the same method, and the preparation conditions are listed in Table 1.

### 2.3. Synthesis of PHEMMA/LIG and PHEMMAC/LIG

The PHEMMA/LIG composite non crosslinked prepared in this work only for the physic-chemical analysis was obtained by the free radical copolymerization of HEMA with MMA in THF using AIBN as an initiator. A defined ratio of HEMA and MMA that was previously degassed and distillated under reduced pressure was dissolved in THF and added to 0.1 wt % of AIBN. A defined amount of LIG was then added to the monomeric solution before starting the polymerization process, which occurred through the same route described in the previous section. A very viscous solution containing the medication and the copolymer was then casted and dried by the same method described in the previous section. The PHEMMAC/LIG, in which the copolymer is crosslinked, was prepared by the same method with only an addition of 37 µL of degassed and distillated EGDMA to the monomeric solution. A jelly solution containing PHEMMA hydrogel and lignocaine was then casted and dried by the same technique. To remove all LIG particles aggregated, deposed, or embedded on the surface of the resulted films, all specimens were washed with water before use. Noting that, the particular reason for choosing exactly 0.12 g of LIG is mainly based on the maximum LIG limit that can be incorporated into the polymer matrix without altering the transparency and the refractive index.

Two series of PHEMMA/LIG and PHEMMAC/LIG drug-carrier systems of different MMA contents were prepared, and the preparation conditions are listed in Table 2.

### 2.4. Characterization

#### 2.4.1. CHN Analysis

The actual content of LIG in the PHEMMAC/LIG composite was determined by CHN elemental analysis using a Perkin Elmer PE 2400 Series II instrument (Perkin Elmer, MA, USA).

#### 2.4.2. H-NMR Analysis

The ^1^H-NMR spectra of poly(methyl methacrylate) (PMMA), poly(2-hydroxy ethyl methacrylate) (PHEMA), and PHEMMA were recorded on a JEOL FX 90 Q NMR instrument (JEOL FX 90 Q NMR, Tokio, Japan) at 500 MHz using DMSO as the solvent.

#### 2.4.3. DSC Analysis

The DSC analysis of the PHEMMA, pure LIG, and PHEMMA/LIG systems and their components was performed on a Shimadzu DSC 60A (Shimadzu, Kyoto, Japan, previously calibrated with indium. Samples weighing between 8 and 10 mg were packed into aluminum DSC pans before being placed in the DSC cell. The samples were scanned from 30 to 200 °C under nitrogen atmosphere at a heating rate of 20 °C·min^−1^. The obtained thermograms revealed that the PHEMMA/LIG system and their pure components did not undergo degradation. Glass transition temperatures *T_g_* were derived accurately from the thermograms as the midpoint in the heat capacity variation with temperature. To eliminate all eventual volatile compounds incrusted in the polymer, such as the solvent and residual monomer, the *T_g_* values were taken from the second run of the DSC process.

#### 2.4.4. Fourier Transform Infrared Analysis

FTIR spectra of the PHEMMA/LIG systems and their components were recorded by a Nicolet 6700 FT-IR, Thermo Scientific (Thermo Scientific, MA, USA). The reflectance spectra of each sample were directly collected between 400 and 4000 cm^−1^ without any treatment. The pure lignocaine was used as a powder, and the composites were used as films prepared by solvent casting.

#### 2.4.5. X-ray Diffraction Analysis

The uniform dispersion of LIG particles in the PHEMMA matrix was analyzed by the X-ray diffraction (XRD) method using an X-ray diffractometer (RigakuDmax 2000) with a Cu anode tube at a voltage/current of 40 kV/40 mA and a generator current of 100 mA. All samples were scanned in the 5°–60° two theta range at a scan rate of 1.0 °C min ^−1^.

#### 2.4.6. Water Content

A known mass of film sample (specimen) was placed in a vacuum oven maintained at 40 °C for 24 hours to extract all traces of moisture incrusted in the polymer matrix. This dried specimen was then weighed on a microbalance Sartorius PT2100, immediately placed in a beaker containing distilled water, and then agitated until the maximum swelling was reached, thus characterizing the swelling equilibrium. To reach this goal, this specimen was removed at an equal interval time, dried on the surface by a paper handkerchief, and then weighed, giving the mass of the wet sample. The equilibrium water content (EWC) in the specimen was calculated based on the mass of the wet specimen, *W_wet_*, and its dry mass, *W_dry_*, according to Equation (1):

(1) $$EWC\left(\%\right)=\frac{W_{wet}-W_{dry}}{W_{dry}}\times 100$$

#### 2.4.7. Mechanical Testing

Young’s modulus measurements of the hydrated specimen were performed on a micromechanical tester Dynac Dalta type (Scientific Instruments, Bradford, UK). Samples measuring 4 cm × 1 cm × 0.2 cm were attached at each end by Luer-Lok handles specifically designed for this purpose and separated at a rate of 0.5%·min^−1^ for 15 s. Tensile force, F (in grams), and elongation of the specimen were calculated from strain (D). The cross-sectional area, S (in mm^2^), was evaluated with precision from the dimensions of the sample measured using an optical microscope. The stress (*P*) applied to the specimen was determined according to the Equation (2):

(2) $$P\left(kPa\right)=\frac{9.8\times F}{S}$$

The apparent elastic modulus (E) of the sample was directly deduced from the slope of the curve, indicating the variation of the content versus elongation, as shown in Equation (3):

(3) $$E\left(kPa\right)=\frac{P}{D}\times 100$$

#### 2.4.8. UV-Visible Analysis

The LIG amounts released from the PHEMMAC/LIG drug-carrier systems were determined by an Evolution 600 UV-visible Spectrophotometer (Thermo Scientific) at 264 nm, corresponding to the maximum absorption of this drug in water. The transparency of the specimens was examined on wet films of the same dimensions (~1 cm × 2 cm × 0.1 cm). These films were placed perpendicular to the path of the beams of UV-visible light. The percent transmittance of each sample was measured against a colorless, transparent glass plate of essentially the same size.

#### 2.4.9. Refractive Indices

For this measurement, the specimen of the copolymer or composite was prepared in a cylindrical form by the solvent casting method. A concentrated solution containing copolymer or composite in THF was placed in a small cylindrical glass capsule. The assembly was then allowed to air dry for 72 h then in the vacuum oven at 40 °C for 24 h to remove any trace of solvent or residual monomers. The capsule was then broken and the specimen was recovered in a cylindrical form. The optical refractive index of these samples was determined using a collimated (1 mm diameter) laser beam illuminated by air in the side of the hydrogel cylinder, and the recording of the optical path realized from the height of the cylinder. The angles of incidence, $\theta_{in},\;$ and refraction, $\theta_{in},$ as well as the index of refraction, *n*, of the samples were calculated using the Snell equation.

(4) $$n=\frac{sin\theta_{in}}{sin\theta_{re}}$$

In this application, three laser lamps of different wavelengths ranging from 405 to 670 nm were used to record the visible spectral range and the value of the refractive index of each specimen was taken by calculating the arithmetic mean of three measurements. The dispersion curve of the refractive index between 400 and 800 nm was adjusted by means of the Cauchy relationship:

(5) $$n\left(\lambda \right)=B+\frac{C}{\lambda^{2}}$$

where B and C are empirical coefficients derived from the fit curve [12].

#### 2.4.10. Cell Viability

An evaluation of the cytotoxicity of the polymers and copolymers was carried out by the 3-(4,5-dimethylthiazol-2-yl)-2,5-diphenyltetrazolium bromide (MTT) test on the L929-type fibroblast cell line according to the Mosmann method [13]. The cells were placed in DMEM supplemented with 10% FBS in a humidity-controlled incubator maintained at 5% CO_2_ by weight at body temperature (37 °C). The cells were trypsinized for 5 min. The cultured cells were incubated with different concentrations, between 10.0 and 100 μg·mL^−1^ for 24 h of PMMA, PHEMA, and PHEMMA in a DMEM/FBS solution. The cells obtained were then washed several times with phosphate-buffered saline (PBS) and then stained with 50 μL of the MTT solution. After 4 h, the medium was separated, and the solution was measured at 570 nm using a microplate reader (Statfax 2100). Sustainability as a percentage was determined according to Equation (6),

(6) $$Viability\;\left(\%\right)=\frac{D_{T}}{D_{C}}\times 100$$

where *D_T_* and *D_C_* were the optical density of the test and control, respectively.

## 3. Results and Discussion

### 3.1. Characterization

#### 3.1.1. CHN Analysis

The results of the actual PHEMMAC/LIG composition obtained by the CHN elementary analysis are grouped in Table 3. As can be seen from these results, 1.05 wt % of the total amount of the LIG was actually incorporated into the copolymer matrix instead of 1.20 wt %. The remaining fraction (0.13–0.15 wt %) which was deposited or slightly embedded on the surface of the specimens was then washed off as indicated in the preparation section. Given this situation, the washing of such specimens that will be the subject of an application in the drug delivery domain must be mandatory.

#### 3.1.2. H-NMR Analysis

**Figure 1:**
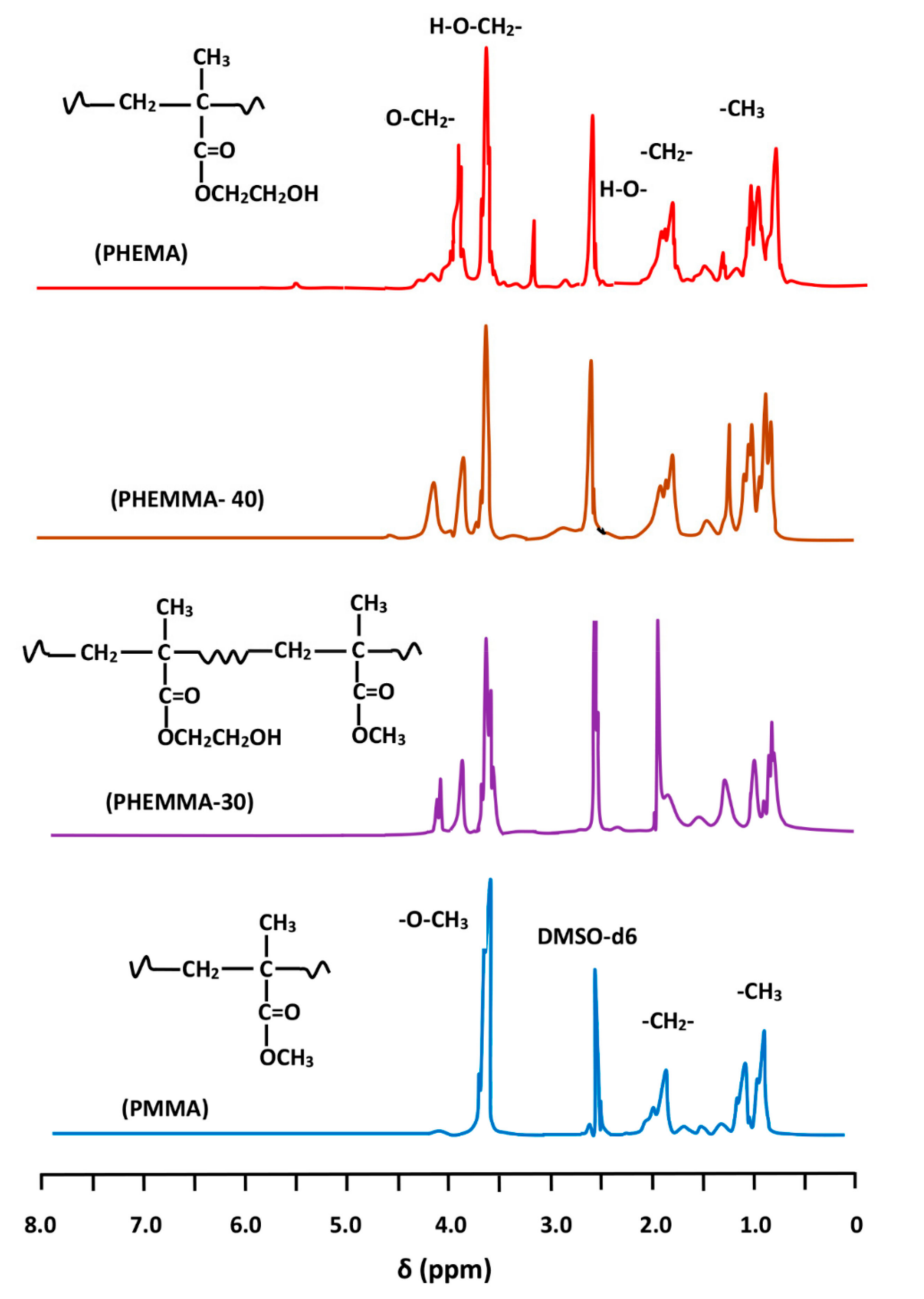
^1^H-NMR spectra of PHEMA, PMMA homopolymers, and PHEMMA copolymers with different compositions.

Figure 1 shows the ^1^H-NMR spectra of PHEMA, PMMA homopolymers, and PHEMMA copolymers with different compositions. The MMA content in the PHEMMA copolymer was determined using the characteristic peak of the HEMA unit at 3.9 ppm assigned to the two protons of the ether group (–O–CH_2_–) and the two protons of the common ethylene group (β) in both the MMA and HEMA units on the main chain. The peaks for these two protons were localized between 1.7 and 2.2 ppm, and the overall MMA content was determined by:

(7) $$MMA\left(mol\%\right)=\left(1-\frac{\delta \left(-O-CH_{2}-\right)}{2\delta \left(-CH_{2}-\right)}\right)\times 100$$

where δ(–OCH_2_–) and δ(–CH_2_–) were the surface area of the signals attributed to the two protons of the –OCH_2_– group and the signal groups of the common –CH_2_– in the two monomeric units (HEMA + MMA), respectively. The obtained results are grouped in Table 3.

#### 3.1.3. DSC Analysis

**Figure 2:**
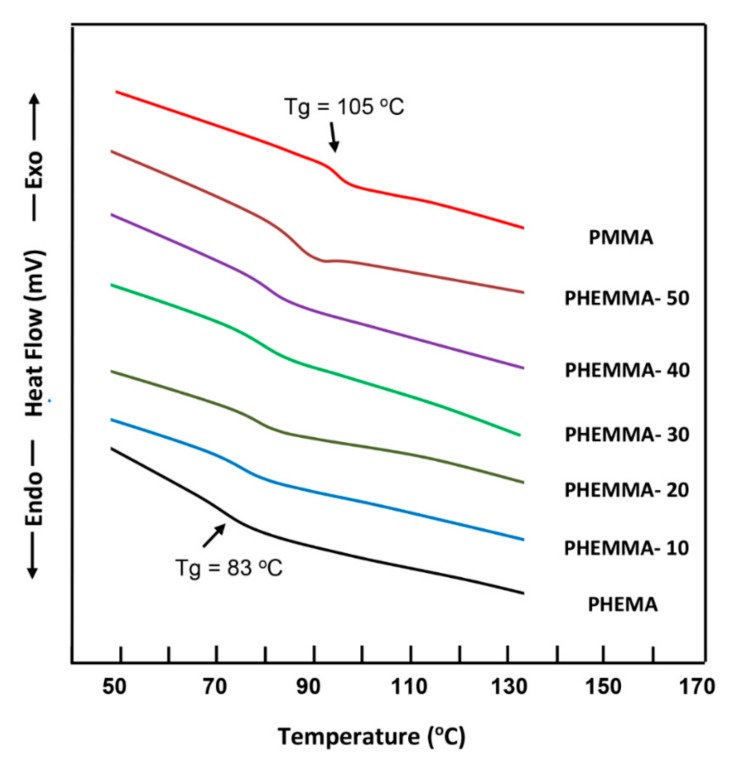
DSC thermograms of pure PHEMA, PMMA, and PHEMMA with different methylmethacrylate (MMA) contents.

**Figure 3:**
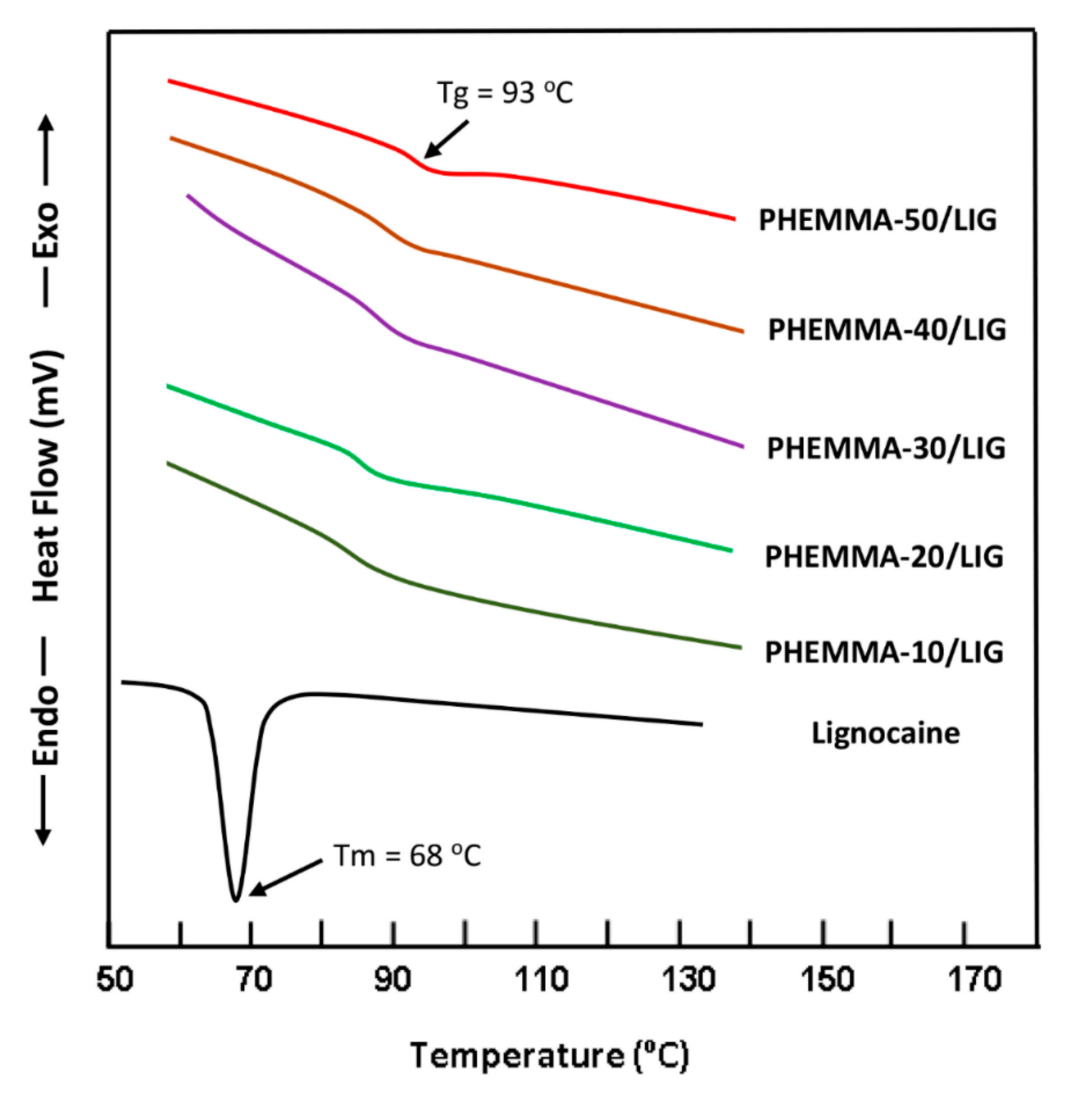
DSC thermograms of PHEMMA/LIG with different MMA contents.

The DSC thermograms of PHEMA, PMMA and PHEMMA copolymers containing different MMA content are shown in Figure 2. The *T_g_* values of homopolymers agreed with those in the literature [14,15]. As seen by the curve profiles, only one glass transition temperature is detected between those of the pure polymers, which shifted towards the glass transition temperature of PMMA when the MMA content varied from 13.26 to 63.32 mol %, indicating the copolymer formation. The thermograms of pure LIG and the PHEMMA/LIG drug-carrier systems with different MMA contents are shown in Figure 3. The total disappearance of the endothermic peak attributed to the melting temperature of LIG at 68 °C [16] indicated that this drug was consistently distributed in the copolymer matrix in its molecular state and not aggregated. The comparison between the thermograms of copolymers of Figure 2 and those of the PHEMMA/LIG systems in Figure 3 revealed a shift in the *T_g_* values toward the low temperatures, as shown in Table 4. This phenomenon can be explained by an increase in chain sliding due to the insertion of the LIG particles between the polymer chains in the plasticizer.

#### 3.1.4. FTIR Analysis

**Figure 4:**
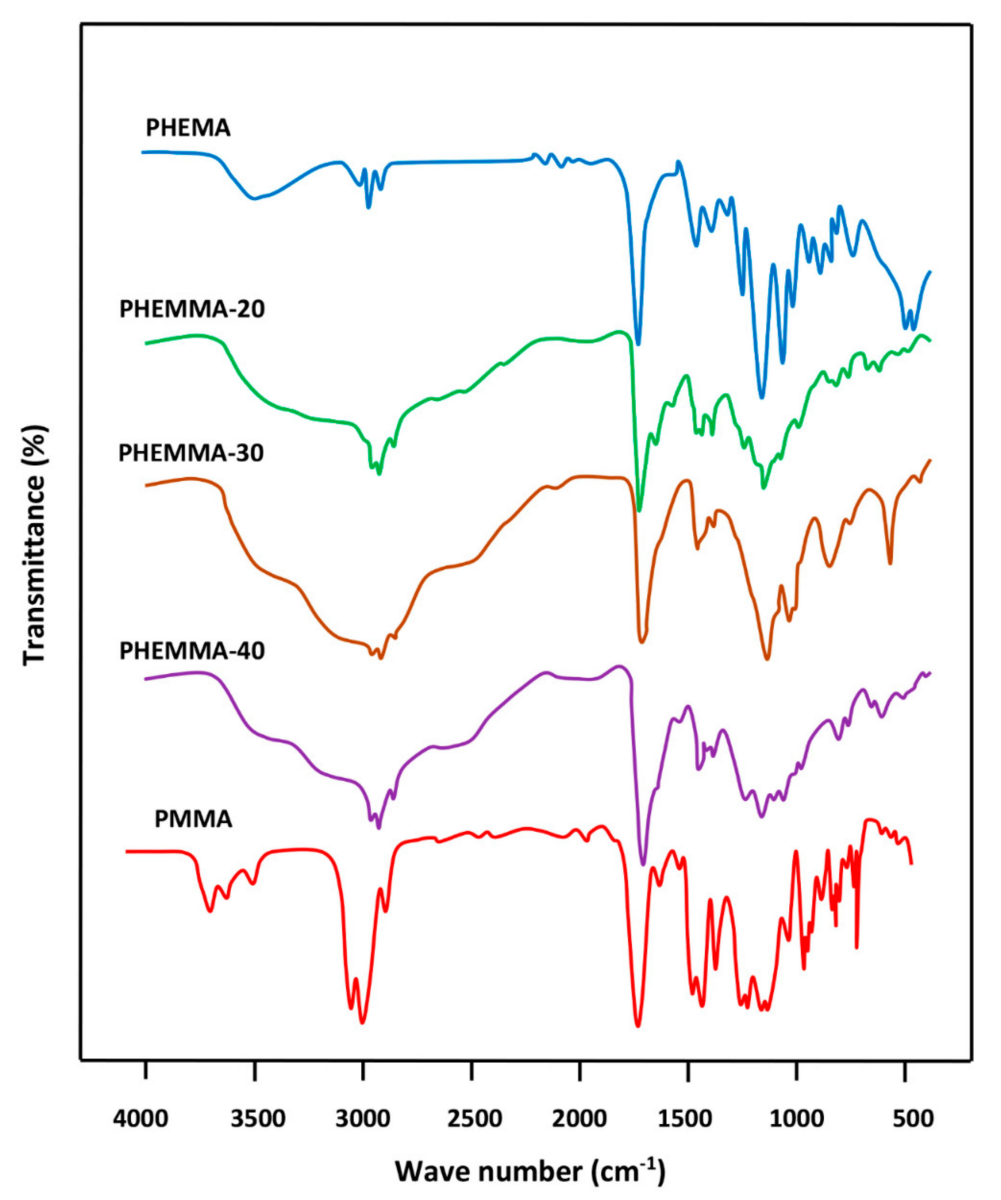
FTIR analysis of PHEMA, PMMA, and PHEMMA with different MMA contents.

**Figure 5:**
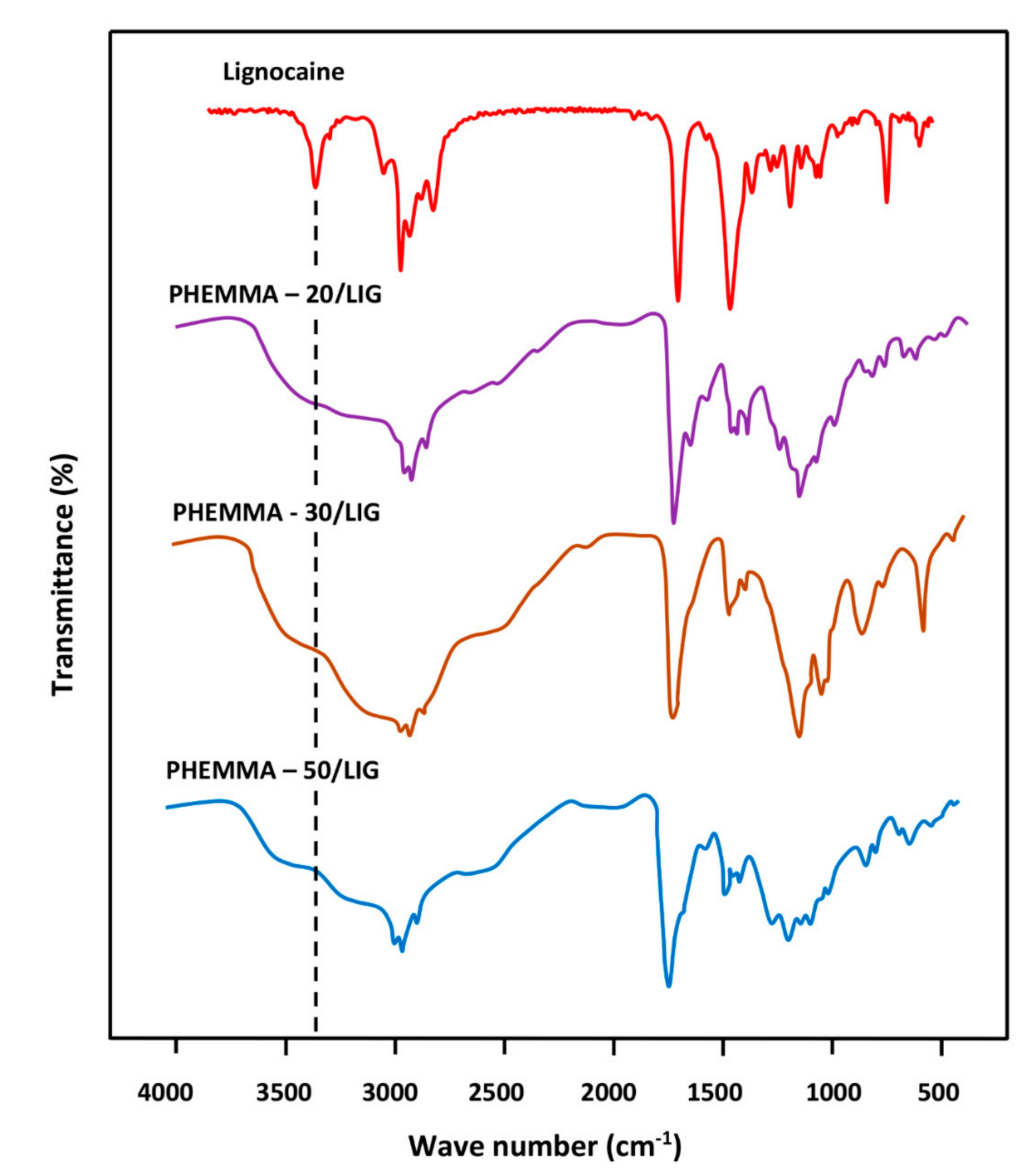
FTIR analysis of LIG, virgin PHEMMA, and PHEMMA/LIG systems with different MMA contents.

The FTIR spectra of PHEMA, PMMA, and PHEMMA with different MMA contents are shown in Figure 4, and the data of the carbonyl absorption bands for comparison are presented in Table 5. A shift in the carbonyl band towards the low wave numbers was observed, and a broad band or a duplet in which its area increased when the MMA content in the copolymer increased. This phenomenon was due to a rearrangement of the interaction forces between the two different carbonyl groups belonging to the two monomeric units and the hydroxyl group of the 2-hydroxyethylmethacrylate unit of the copolymer. The broad band localized between 3125 and 3690 cm^−1^ was attributed to the –OH group, which covers five absorption bands at 3666 cm^−1^ assigned to the –OH (trans), 3624 cm^−1^ (–OH, gauche), the hydrogen bond with –C=O, the hydrogen bond of the dimer (–OH…–OH), and the first overtone of the –C=O stretching group [17]. Figure 5 shows the FTIR spectra of pure LIG and the PHEMMA/LIG with different HEMA contents. The pure LIG spectrum presented a –C=O stretching group at 1722 cm^−1^ and a –N–H stretching group at 3451 and 3385 cm^−1^ [18]. The comparison of the spectra in these two figures reveals that the addition of 1.05 wt % of LIG to the copolymer led to a significant shift in the absorption band of the carbonyl group, which decreases with an increasing MMA unit in the copolymer. This finding was likely due to the hydrogen bond interaction created between the hydroxyl group of the HEMA unit and the carbonyl of LIG. Indeed, the addition of the MMA unit in the copolymer caused a decrease in the density of the hydroxyl group on the polymer chain, resulting in a decrease in the density of the hydrogen bonds. This was also confirmed on the side of the absorption bands of hydroxides, through the change of the form of the absorption band. These results suggested the presence of LIG in the polymer in its molecular state.

#### 3.1.5. X-ray Diffraction Analysis

**Figure 6:**
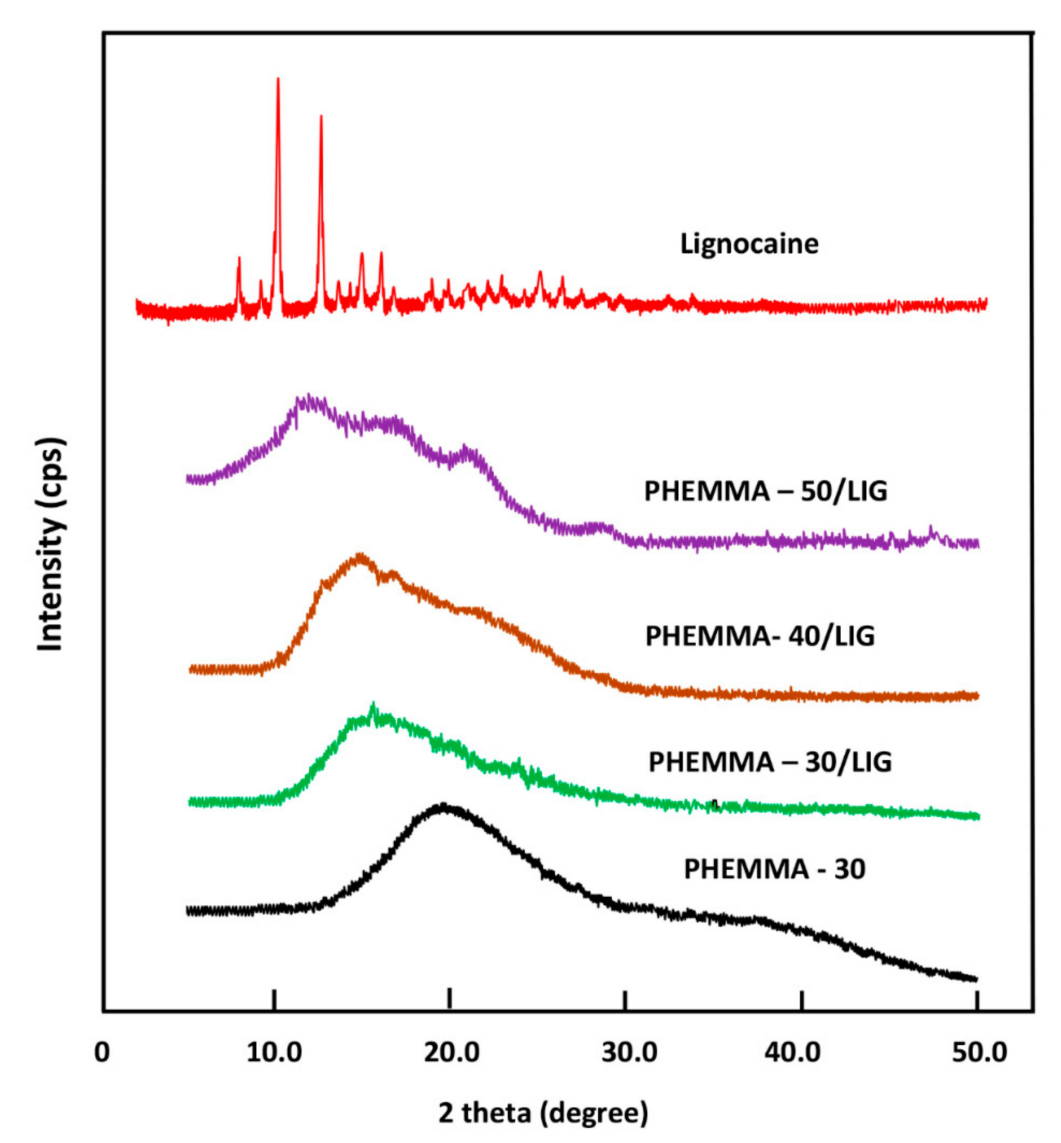
XRD patterns of pure LIG, PHEMMA, and PHEMMA/LIG systems with different MMA contents.

The XRD analysis of the LIG powder, shown in Figure 6, revealed several sharp absorption peaks. The main peaks localized at 7.8, 10, 12.5, 15, and 16.2 2θ, indicating the crystalline nature of this medication. However, no sharp peaks were observed in the PHEMMA patterns, indicating the amorphous nature of this copolymer at any co-monomers composition. The spectra belonging to the PHEMMA/LIG systems did not reveal any sharp signals indicative of the crystallinity of this system, indicating that these composites have amorphous structures. These results suggested that LIG was uniformly distributed in the copolymer matrix in its molecular state, as consistent with the results obtained by FTIR. 

#### 3.1.6. Water content at equilibrium, EWC

**Figure 7:**
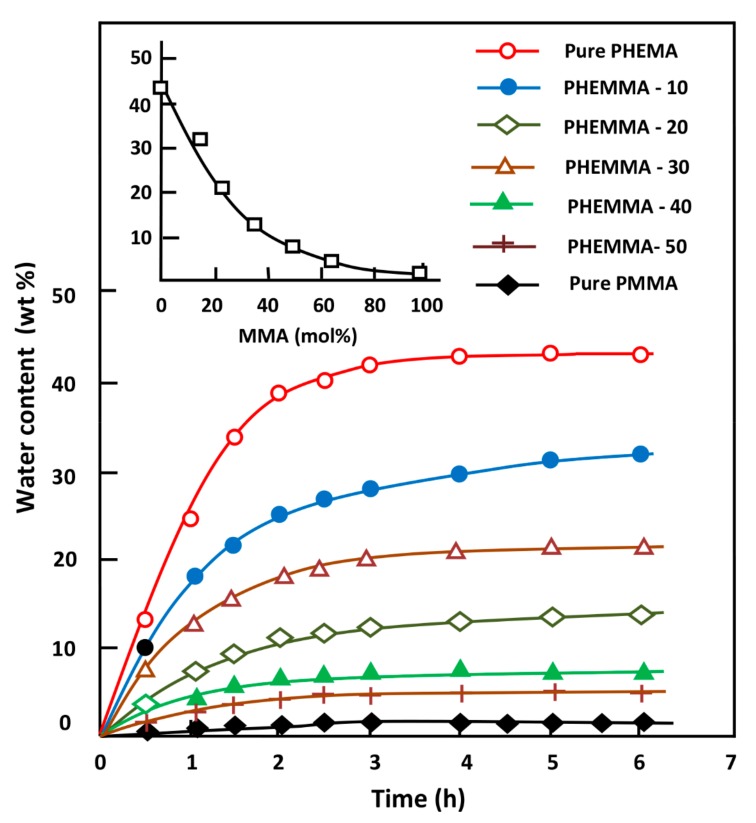
Variation of the equilibrium water content (EWC) in the PHEMMAC specimens and in their components versus time and the MMA content (top left).

The EWC in the PHEMA, PMMA homo-polymers, and PHEMMAC with different compositions was estimated using Equation (1), and the obtained results are illustrated in Figure 7; the EWC values are given in Table 4. The swelling at saturation was reached during the 4 h swelling process in practically all specimens; 43 wt % of water was absorbed by the pure PHEMA, and 1.3 wt % was absorbed by the pure PMMA. As expected, the incorporation of the MMA unit (hydrophobic monomeric unit) into PHEMA significantly reduced the degree of swelling of this polymeric material following an exponential curve, as shown in the top left of Figure 7. Indeed, 63.32 mol % of MMA in the copolymer reduced the EWC of pure PHEMA by 3X. Controlling the swelling degree of a polymer carrier was considered a working tool that potentially controlled the parameters governing the dynamics of the drug delivery process, such as the amount of drug released, the release rate, and the release duration.

#### 3.1.7. Mechanical Testing

The values of the apparent Young’s modulus, E, of the two pure homo-polymers and their crosslinked copolymers (PHEMMAC) with different MMA contents determined from Equation (3) are listed in Table 6. These data indicated an E value of 17 ± 4 kPa for pure PHEMA and 32,000 ± 43 kPa for PMMA, which were close to those reported in the literature [19,20]. The apparent Young’s modulus dramatically increased when the MAA content in the copolymer increased, thus decreasing the flexibility of the PHEMA. This finding was expected because of the large differences between the E values of the pure component and those of EWCs. The comparison of the EWCs and the apparent Young’s modulus with those of commercial lenses such as Phemefilcon which is fabricated from copo (2-hydroxyethyl methacrylate-co-ethoxyethyl methacrylate-co-methacrylic acid) ter-polymer and manufactured by Ciba Vision company (USA) indicates a good correlation with 38 wt % and 300 KPa (measured central zone thickness (MCZT)). Measured with 10 mm diameter probe micrometer [21].

#### 3.1.8. Transparency

**Figure 8:**
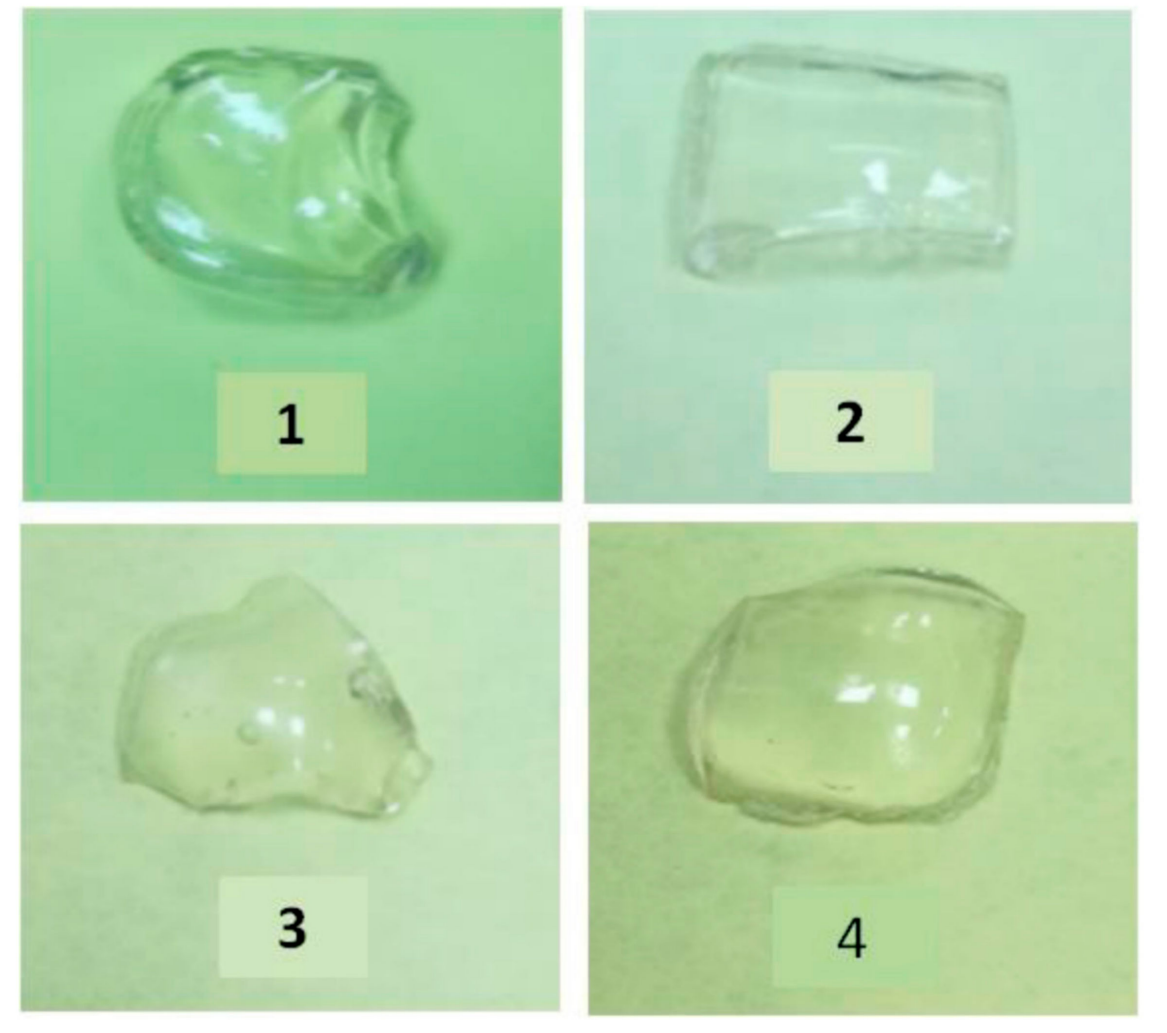
Photographs of wet PHEMMAC specimens containing: (**1**) 13.26 wt %; (**2**) 22.18 wt %; (**3**) 33.21 wt %; (**4**) and 63.32 mol % MMA.

**Figure 9:**
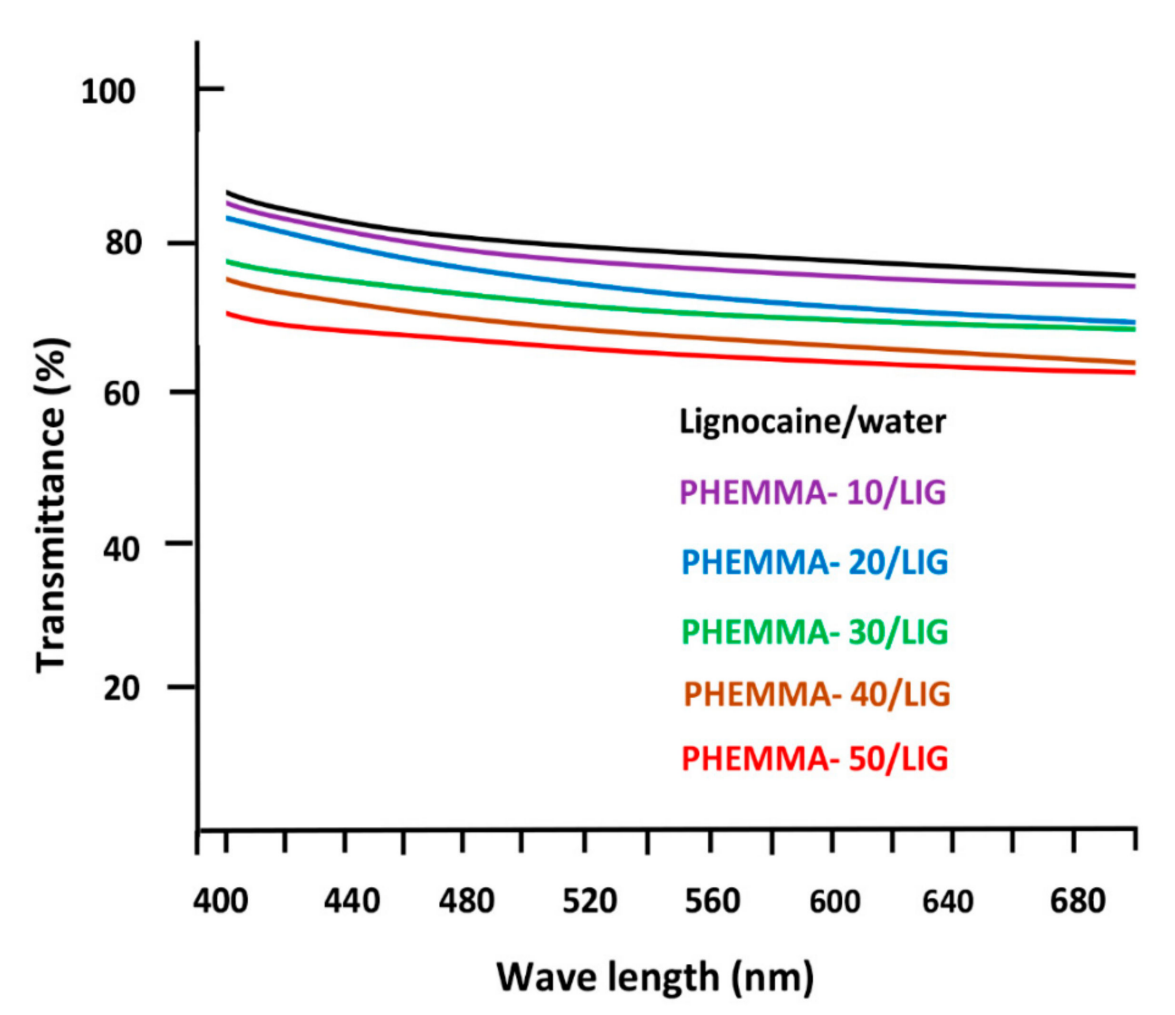
Spectra of the wet PHEMMAC/LIG with different MMA contents: (**A**) LIG/water; 13.26 mol %; (**B**) 22.18 mol %; (**C**) 33.21 mol %; (**D**) 48.56 mol %; and (**E**) 63.32 mol % recorded in the visible light domain (400–700 nm).

Figure 8 shows the photos of wet PHEMMAC/LIG specimens with different compositions and different water contents examined directly by ocular vision. As seen in these photos, all samples were colorless, transparent, and homogeneous. These observations were supported by the UV-visible analysis in the visible light domain (400–700 nm). Indeed, as shown in Figure 9, more than 65% of the transmittance was noted and decreases slowly with increasing wave numbers. The maximum transmittance (84%) was reached with the specimen containing 13.26% MMA.

According to the Bouguer et Lambert’s Law, transmittance through a thickness of 0.1 cm is considered as an excellent transparency. This finding also suggested the absence of any aggregated LIG particles in the polymer matrix, which could reduce the transparency of the polymeric specimen.

**Figure 10:**
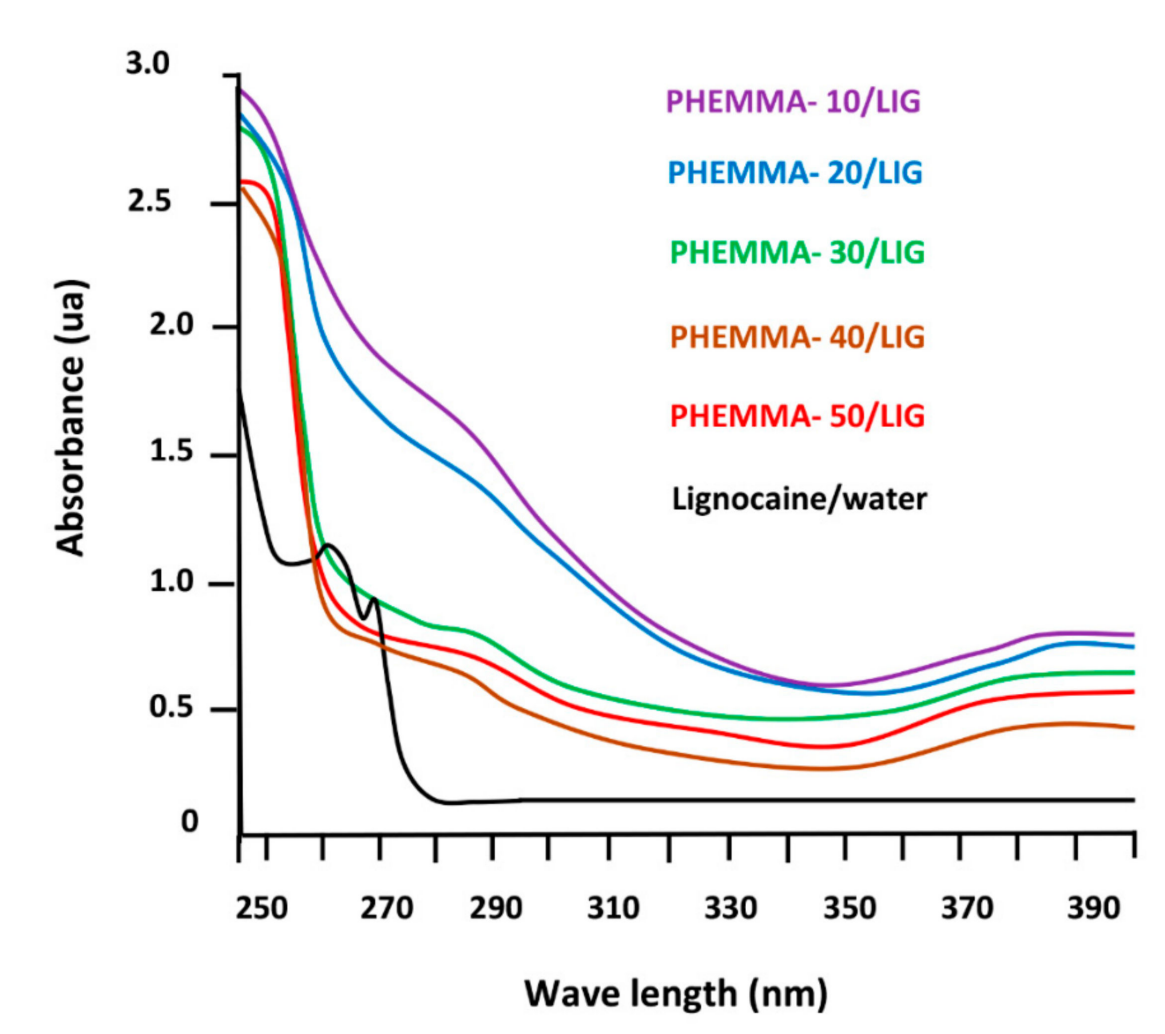
Spectra of the wet PHEMMAC/LIG with different MMA contents: (**A**) LIG/water; 13.26 mol %; (**B**) 22.18 mol %; (**C**) 33.21 mol %; (**D**) 48.56 mol %; and (**E**) 63.32 mol % recorded in the visible light domain (240–400 nm).

Figure 10 shows the spectra of the PMMA, PHEMA, and PHEMMAC/LIG systems with different MMA contents recorded in the UV light zone (240–400 nm). The pure LIG spectrum showed two absorption bands at 262 and 270 nm, characterizing the electronic transitions of this compound. The large absorption band centered at 278 nm overlapped with the different transitions assigned to the carbonyl common to the methacrylate units of the copolymer. Knowing that the percentage of this pharmacon was very low compared to that of the copolymer, the transition bands belonging to this product were also covered by this large band.

#### 3.1.9. Refractive Indices

The determination of the optical refractive index is essential in the characterization of a material for potential use as contact lenses in the medical field. According to Varikooty et al. [22], a relatively high refractive index can reduce the lens thickness and thereby significantly improves the wearer’s comfort. Thus, the refraction indices of the samples were determined by measuring the refraction of the light in each material prepared and the results obtained are illustrated in Table 7. As can be seen from these data, no sensible change in the refractive index is observed with changing the composition in the co-monomeric units of the virgin PHEMMAC. These results agree with those of the literature for comparable materials [23,24]. The addition of 1.05 wt % of LIG to this copolymer material slightly affects this optical parameter, in which a slight shift toward the high refractive index is observed. This finding suggests that the LIG is really uniformly distributed in the PHEMMAC matrix in its molecular state. Contrarily, the presence of eventual aggregated LIG particles can easily deviate the optical path of the laser beam, which is not our case here.

#### 3.1.10. Cell Viability

**Figure 11:**
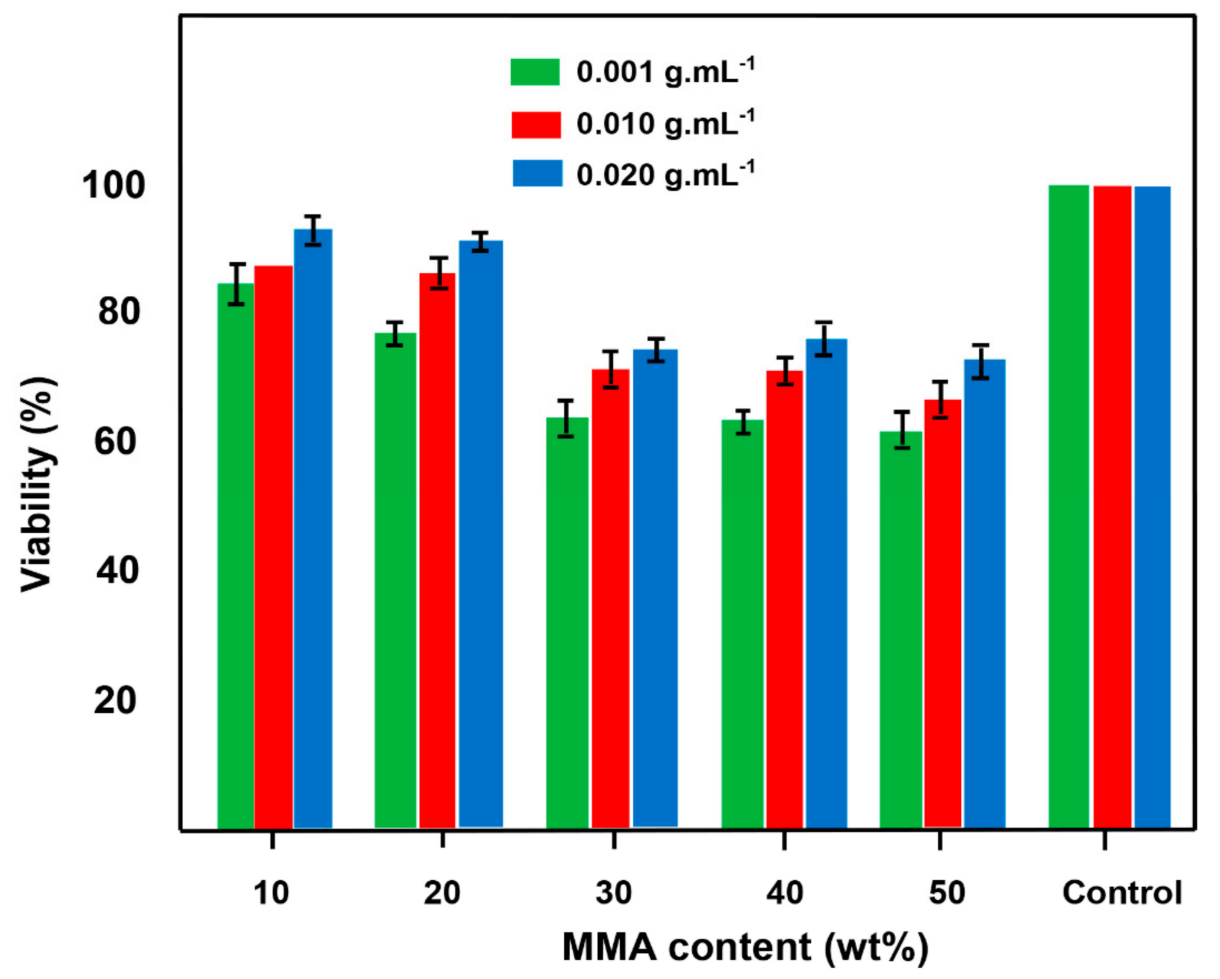
The diagram of the in vitro cytotoxicology text of PHEMMAC film specimens on L929 cells after 24 h of incubation.

The results of the cytotoxicity test of the prepared copolymer determined by the MTT assay through the percentage of the cell viability obtained by Equation (1) are given in Figure 11. As shown in these diagrams, cell viability increased when the concentration of the copolymer in the DMEM/FBS solution increased. The viability percentage of this system decreased slowly when the MMA content in the copolymer increased; 62–80% and 76–93% viability (38–20% and 24–7% toxicity) were demonstrated for 0.001 and 0.020 g·mL^−1^ of PHEMMAC copolymer, respectively. The cell viability was above 62% for all concentrations tested. This finding seems indicate no significant toxicity of the PHEMMAC carrier and demonstrated that PHEMMAC was a safe drug-carrier system.

### 3.2. In Vitro LIG Release

#### 3.2.1. Kinetics Release of LIG

**Figure 12:**
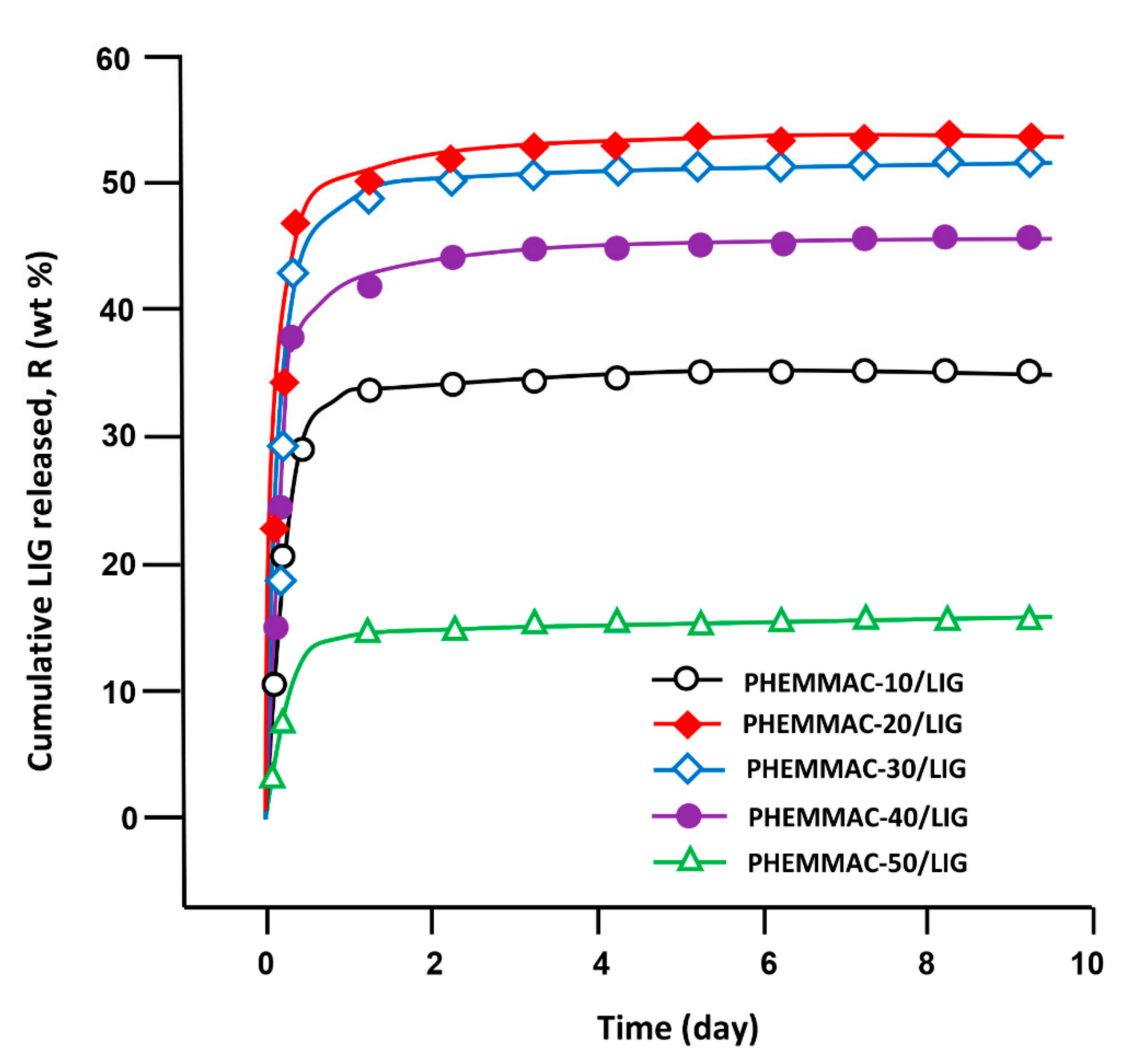
Cumulative LIG released from the PHEMMAC drug-carrier systems.

The cumulative LIG released from PHEMMAC/LIG hydrogel with different MMA contents in neutral media pH was determined at 37 °C during 9 days, and the results obtained with Equation (8) are plotted in Figure 12.

(8) $$R(wt\%)=\frac{m_{t}}{m_{o}}\times 100$$

where *m_o_* and *m_t_* were the initial mass and mass at time *t* of LIG in the drug-carrier system, respectively. As seen from the curve profiles, the percentage of LIG released over time followed a logarithmic growth, in which 16 and 54 wt % of the total amount of drug was released depending on the MMA content incorporated in the drug-carrier system. These patterns also revealed that 85 to 90 wt % of LIG was cumulatively released during the first five hours, depending on the MMA content in the PHEMMAC. This phenomenon was due to the high difference between the free enthalpy of dissolution (ΔG_d_) inside and outside of the polymer matrix until equilibrium was reached.

From these curve profiles, it was also revealed that the maximum release of LIG was achieved with 22.18 mol % of MMA (PHEMMAC-20/LIG). Indeed, this system released more than 54 wt % of this medication during all release periods. The lower dynamic release was observed with the drug-carrier system containing 63.32 mol % of MMA (PHEMMAC-50/LIG), which was capable of releasing only 16 wt % of LIG during the same duration. This finding reflected the capacity of the MMA incorporated in the carrier to reduce the dynamic release of this drug in a neutral pH media, possibly causing a reduction in the swelling degree of the polymer hydrogel. This was likely due to the increase of the hydrophobic regions in the PHEMMAC copolymer matrix; however, the release with 13.26 mol % of MMA was governed differently.

#### 3.2.2. Diffusion Behavior of Lignocaine

**Figure 13:**
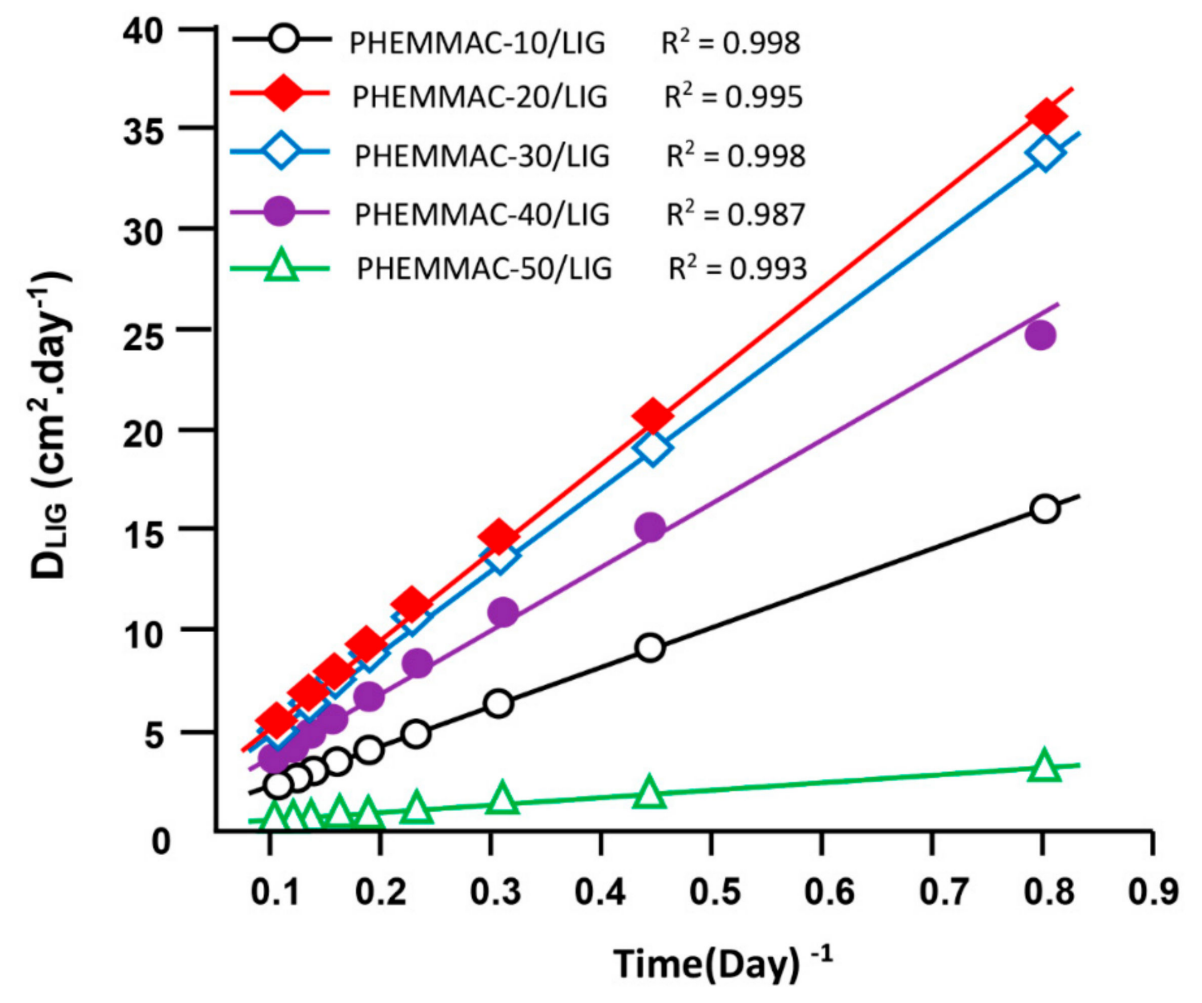
Variation of the diffusion coefficient of lignocaine through the PHEMMAC/LIG drug-carrier systems with different MMA contents versus the inverse of time.

According to Lin et al. [25], the diffusion of a drug through a polymeric material follows the Fickian model if the cumulative drug released in a medium does not exceed 60 wt % of the total drug amount loaded in the polymer matrix. In this condition, the value of the diffusion coefficient, *D_LIG_* was calculated from Equation (9) [26,27,28,29,30,31]:

(9) $$D_{LIG}=\frac{0.198\times d^{2}}{t}\times {\left[\frac{m_{t}}{m_{o}}\right]}^{2}$$

where *d* is the thickness of the film specimen. The *D_LIG_* value was determined when the permanent regime was reached, and therefore, all LIG particles deposed or pressed on the surface of film surface were totally removed by washing in water. In these conditions, the curve profiles of *D_LIG_* versus time were meaningful and reflected the dynamics of LIG released in the media inside the polymer matrix. The variation of *D_LIG_* versus the inverse of time of the PHEMMAC/LIG drug-carrier system with different MMA contents, as calculated from Equation (9), and the experimental data of Figure 12 are shown in Figure 13. As shown from these curve profiles, straight lines were obtained for each HEMA/MMA composition. This finding indicated that the diffusion of LIG through the PHEMMAC hydrogel followed the Fickian model. This finding also indicated that the permanent regime of the dynamic release was reached. In light of these results, it was possible to determine the second stage of the release process, which was generally localized between 8 h and 9 days.

#### 3.2.3. Effects of the MMA/HEMA Composition

**Figure 14:**
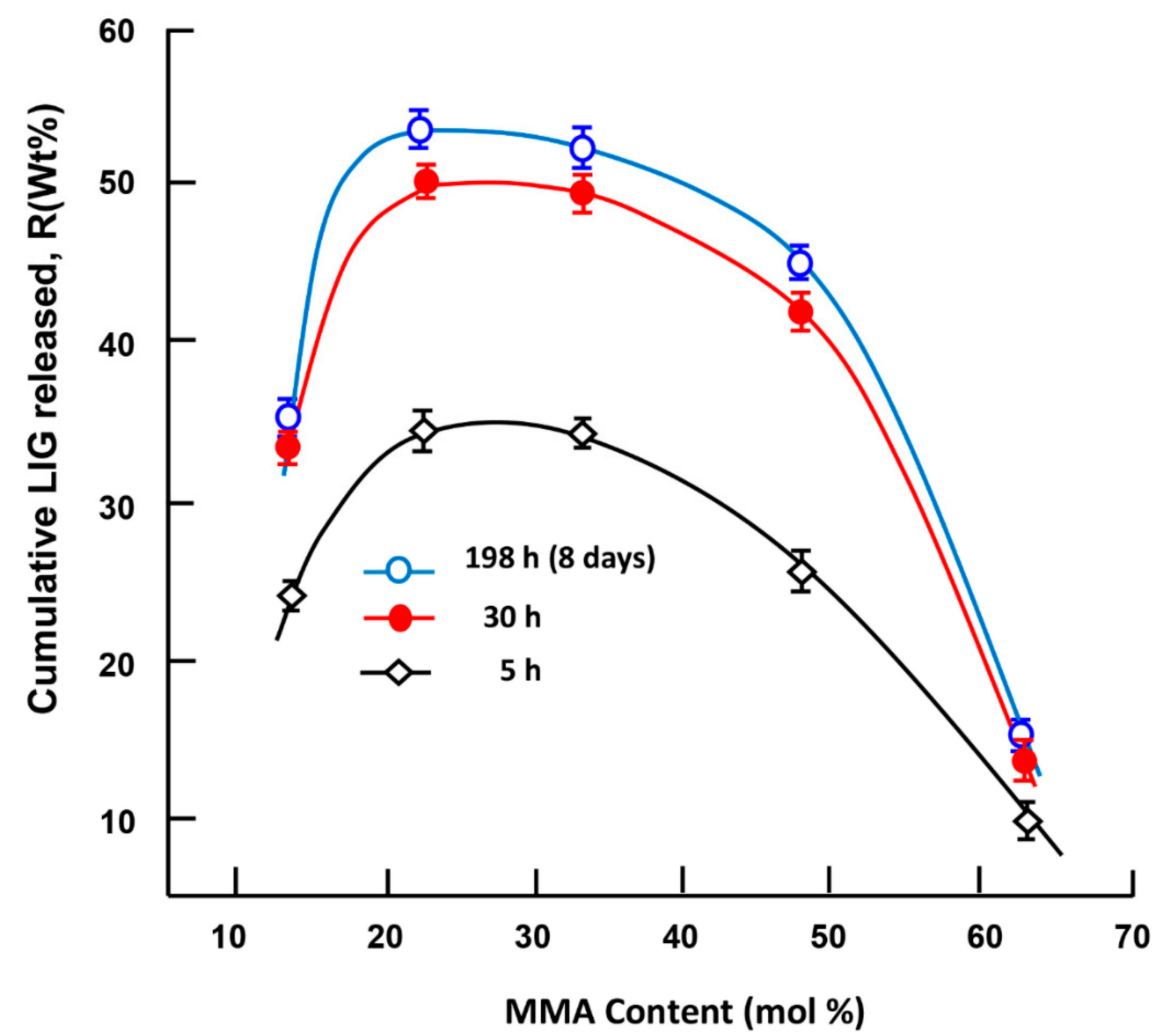
Variation of the cumulative LIG released from the PHEMMAC/LIG system versus the MMA content taken during 5, 30, and 198 h of the release process.

Figure 14 shows the effects of the MMA content incorporated in the PHEMMAC carrier on the dynamic release of LIG in neutral pH media during 5 h, 30 h, and 198 h (8 days) of the release process. The dynamic release followed the same pattern for all time-points, in which the cumulative release of LIG dramatically increased; the maximum LIG release occurred in the copolymer containing 22.18 mol % of MMA (PHEMMAC-20/LIG), and the lowest amount was released in the copolymer containing 48 mol % of MMA. As expected, LIG release decreased when the MMA/HEMA composition increased over 23 mol % of MMA in the PHEMMAC, which can be attributed to a reduction of the swelling degree of the copolymer caused by the hydrophobic characteristics of MMA incorporated in the carrier system. In this situation, the water content inside the hydrogel was dramatically reduced, and there was not enough water for the dissolution of this medication.

#### 3.2.4. Effects of the water content at equilibrium

**Figure 15:**
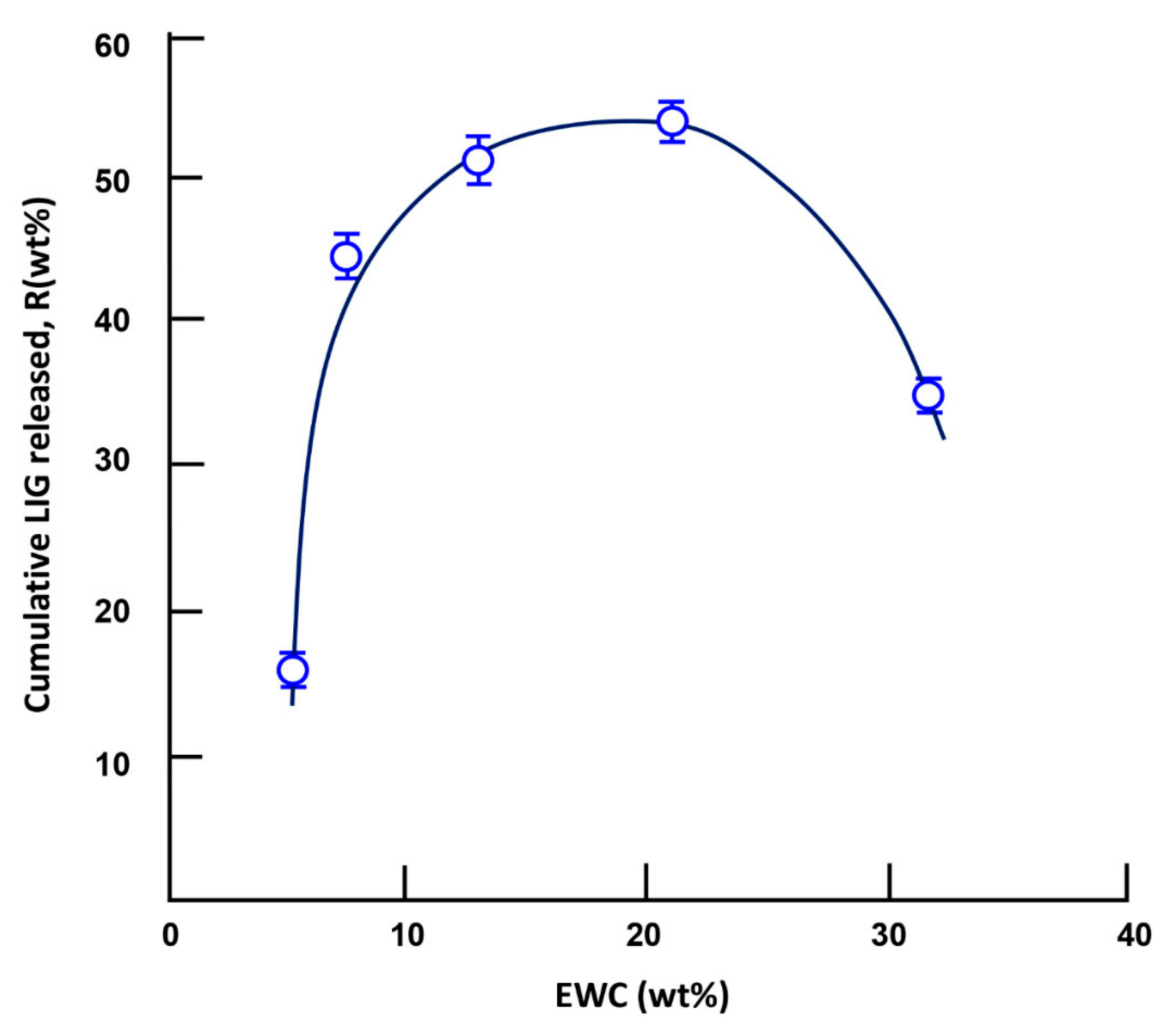
Variation of the cumulative lignocaine released versus the water content at equilibrium during the 3-day release process.

The dynamic release of a drug from a drug-carrier system does not only depend on the water–drug solubility, pH of the media, drug/carrier composition, and drug and carrier structures, but also on other determining parameters, such as the swelling degree of the polymer used as a carrier. Different authors have investigated this parameter [32,33,34,35,36,37]; however, the mechanism is still poorly understood due to the multiple factors that intervene at the same time. For example, Khalid et al [32] reported the effects of crosslinking on swelling and on the drug release behavior of site-specific lactulose from poly (methyl methacrylate-*co*-itaconic acid) (P(MMA/IA)) hydrogels. The dynamic release of lactulose decreased in neutral pH and stabilized at low pH when the swelling degree of the copolymer increased. Wan et al. [33] investigated the Ibuprofen/hydroxy propyl methylcellulose (HPMC) drug-carrier system, in which the effect of the swelling degree was widely studied. It was revealed than an inverse relationship exists between the drug release rate and the matrix swelling rate. In this study, the effect of the swelling degree, characterized by the EWC, on the cumulative amount of LIG released is traced in Figure 15. As observed in the curve profile, the cumulatively released LIG reached a maximum when the EWC was between 13.26 and 22.18 wt %. An increase in the release dynamic at an EWC less than 13.26 wt % can be attributed to an increase of the water dissolution of lignocaine, which increases with the water content in the polymer matrix; a decrease at an EWC of less than 22.18 wt % was likely caused by competition between the water drug dissolution and the hydrogen bond (H–O…H–N) interaction between the hydroxyl of the HEMA unit and the secondary amine group of LIG, which increased with the HEMA content in the copolymer.

#### 3.2.5. Performance of the PHEMMAC/LIG Drug-Carrier System

The kinetics of LIG release evaluated in this study were based on the data of the instantaneous release rates of this drug from the PHEMMAC/LIG drug-carrier systems taken from the slopes of the linear portions of the kinetic curves shown in Figure 12. According to these curve profiles, the presence of two principal stable zones for each MMA/HEMA composition was revealed, as listed in Table 8. For example, the PHEMMAC/LIG system containing 22.18 mol % of MMA (PHEMMAC-20/LIG) has its first zone at 5 h of the release process. This stable zone was short and characterized by a cumulative LIG released of 46.0 wt % with a release rate of 9.4 wt %·h^−1^. This step was followed by a larger zone localized between 18 and 168 h, in which 4.80 wt % of the total LIG amount was released with a release rate of 0.033 wt %·h^−1^. According to the criteria based on the maximum drug amount uniformly released with an adequate release rate during the longest period, the PHEMMAC/LIG systems containing 22.18 and 33.21 mol % of MMA incorporated in the copolymer matrix had the best performance. Indeed, both systems were capable of uniformly releasing the maximum percentage of LIG, 50.8 and 52.0 wt %, respectively, in neutral pH medium during the same period (7 days). The drug-carrier system containing 33.21 mol % of MMA uniformly released a total of 47 wt % of LIG during a period of 5 h with a release rate of 6.5 wt %·hour^−1^ and released 5 wt % during a 5.5-day period with a moderate release rate of 0.033 wt %·hour^−1^. Overall, the best copolymer drug-release system was the PHEMMAC/LIG systems containing 22.18 and 33.21 mol % of MMA.

## 4. Conclusions

The present work was only a pilot study. It demonstrated the feasibility of a synthesized particle-laden hydrophobic/hydrophilic based contact lens that can deliver drugs at a therapeutic dosage for more than a week. The characterization of the prepared drug-carrier system by FTIR, DSC, and XRD revealed that lignocaine was uniformly distributed in the copolymer matrix in its molecular state. The water content in the prepared material at equilibrium decreased dramatically with increasing MMA content in the copolymer, leading to an increase in the apparent Young’s modulus. The transparency test of these materials revealed a maximum transmittance (84%) with the specimen containing 13.26% of MMA. The addition of 1.05 wt % of LIG to this copolymer material slightly affects this optical property of PHEMMAC used as carrier. The cytotoxicity test, using the MTT method, revealed no significant toxicity of the PHEMMAC carrier; thus, this system can be used as safe candidate in drug-carrier system. The diffusion of lignocaine through the copolymer matrix followed the Fickian model, and the dynamic release can be easily controlled through the hydrophilic/hydrophobic characteristics of the MMA/HEMA ratio in the copolymer. The drug release results obtained from the in vitro study of this system in a neutral pH l aqueous medium have been satisfactory and promising. By varying the MMA/HEMA co monomer ratio and the amount of water absorbed by the system, this copolymer can consistently deliver adequate amounts of lignocaine over a long period (>7 days) uniformly, thus minimizing the excess that is usually wasted when administered by the usual method. The limits of use of such a drug-carrier system for application as a drug-contact lenses system must meet, inter alia, the following criteria: (i) the concentration of LIG in the copolymer must not exceed 1.05 wt % in order to not alter the desired optics properties; (ii) the MMA co-monomer composition in the copolymer must not fall outside the range prescribed by this study (10–50 wt %) because a lower quantity leads to an increase in swelling degree (water content) due to the hydrophilic character of the HEMA, and consequently the release will be more rapid and this is not desirable in the field of drug release by the lenses. A quantity greater than 50 wt % considerably reduces this phenomenon due to the hydrophobic character of the MMA.

## Figures and Tables

**Table 1:** Preparation conditions of poly(2-hydroxyethylmethacrylate-co-methylmethacrylate) (PHEMMA) copolymers.

System PHEMMA	MMA (g)	HEMA (g)	AIBN (mg)	THF (mL)
PHEMMA-10	1.0	9.0	10	20.0
PHEMMA-20	2.0	8.0	10	20.0
PHEMMA-30	3.0	7.0	10	20.0
PHEMMA-40	4.0	6.0	10	20.0
PHEMMA-50	5.0	5.0	10	20.0

**Table 2:** Preparation conditions of PHEMMA/lignocaine (LIG) systems.

System PHEMMA/LIG	MMA (g)	HEMA (g)	AIBN (mg)	LIG (g)	LIG (wt %)
PHEMMA-10/LIG	1.0	9.0	10	0.12	1.20
PHEMMA-20/LIG	2.0	8.0	10	0.12	1.20
PHEMMA-30/LIG	3.0	7.0	10	0.12	1.20
PHEMMA-40/LIG	4.0	6.0	10	0.12	1.20
PHEMMA-50/LIG	5.0	5.0	10	0.12	1.20

**Table 3:** Compositions of the poly(2-hydroxyethyl methacrylate-co-methyl methacrylate) crosslinked (PHEMMAC)/LIG system determined by CHN and ^1^H-NMR.

System	Element (CHN)				HEMA (wt %)	MMA (wt %)	LIG (wt %)
	C (wt %)	H (wt %)	O (wt %)	N (wt %)	^1^H-NMR		CHN
PHEMMAC-10/LIG	56.23	7.73	35.80	0.23	85.67	13.21	1.05
PHEMMAC-20/LIG	56.53	7.75	35.48	0.24	78.21	20.67	1.06
PHEMMAC-30/LIG	56.92	7.78	35.05	0.24	68.34	30.54	1.05
PHEMMAC-40/LIG	57.41	7.81	34.52	0.25	57.12	41.76	1.07
PHEMMAC-50/LIG	57.89	7.84	34.00	0.26	46.56	52.32	1.07

**Table 4:** Comparison between the *T_g_* values of the PHEMMA copolymers and those of the PHEMMA/LIG systems.

Polymer and Copolymer	*T*_g_ (°C)	PHEMMA/LIG Composite	*T*_g_ (°C)
PHEMA	83	-	-
PHEMMA-10	86	PHEMMA-10/LIG	84
PHEMMA-20	88	PHEMMA-20/LIG	86
PHEMMA-30	90	PHEMMA-30/LIG	88
PHEMMA-40	92	PHEMMA-40/LIG	90
PHEMMA-50	95	PHEMMA-50/LIG	93
PMMA	105	-	-

**Table 5:** FTIR absorption bands of carbonyl and hydroxyl groups in PHEMMA, PHEMMA/LIG systems, and their components.

System	υ (C=O) (cm^−^^1^)	υ (O–H) (cm^−^^1^)	System	υ (C=O) (cm^−^^1^)	υ (O–H) (cm^−^^1^)
PMMA	1732	-	Lignocaine	1722	3670–3125
PHEMMA-10	1730	3650–3125	PHEMMA-10/LIG	1724	3672–3125
PHEMMA- 20	1728	3650–3130	PHEMMA- 20/LIG	1724	3675–3125
PHEMMA- 30	1727	3650–3135	PHEMMA- 30/LIG	1725	3700–3125
PHEMMA- 50	1727	3690–3145	PHEMMA-50/LIG	1726	3725–3125

**Table 6:** Water content at equilibrium and the apparent Young’s modulus of the pure homo-polymers and the PHEMMAC copolymer specimens with different compositions.

System	EWC (wt %)	Elastic Modulus (kPa)
PHEMA	43.2 ± 0.2	17 ± 4
PHEMMA-10	32.0 ± 0.2	123 ± 12
PHEMMA-20	21.4 ± 0.3	317 ± 11
PHEMMA-30	13.7 ± 0.4	782 ± 25
PHEMMA-40	8.3 ± 0.5	1880 ± 25
PHEMMA-50	5.5 ± 0.5	25,058 ± 32
PMMA	1.3 ± 0.5	32,000 ± 43

**Table 7:** Comparative table of the refraction indices values measured for the virgin PHEMMAC and PHEMMAC/LIG systems with different MMA contents.

Copolymer	Refractive Index at 37 °C			Composite	Refractive Index at 37 °C		
λ (nm)	405	532	670	λ (nm)	405	532	670
PHEMA	1.5220	1.5125	1.4817	-	-	-	-
PHEMMAC-10	1.5205	1.5014	1.4760	PHEMMAC-10/LIG	1.5256	1.5082	1.4783
PHEMMAC-20	1.5175	1.4801	1.4712	PHEMMAC-20/LIG	1.5200	1.4862	1.4734
PHEMMAC-30	1.5140	1.4783	1.4567	PHEMMAC-30/LIG	1.5183	1.4812	1.4583
PHEMMAC-40	1.5032	1.4619	1.4421	PHEMAC-40/LIG	1.5112	1.4657	1.4465
PHEMMAC-50	1.4913	1.4504	1.4326	PHEMMAC-50/LIG	1.5002	1.4552	1.4382
PMMA	1.4878	1.4434	1.4333	-	-	-	-

**Table 8:** Stable zones and instantaneous release of lignocaine from the PHEMMAC/LIG drug-carrier systems with different MMA/HEMA compositions.

Drug-Carrier System	Stable Zone (h)	LIG Released (wt %)	Release Rate (wt %.h^−1^)
PHEMMAC-10/LIG	0–5 5–168	33.50 ± 0.15 0.25 ± 0.08	6.50 ± 0.03 0.002 ± 0.001
PHEMMAC-20/LIG	0–5 18–168	46.00 ± 0.25 4.80 ± 0.32	9.40 ± 0.05 0.03 ± 0.01
PHEMMAC-30/LIG	0–5 36–168	47.00 ± 0.25 5.00 ± 0.32	9.40 ± 0.05 0.04 ± 0.01
PHEMMAC-40/LIG	0–5 120–168	7.80 ± 0.15 1.61 ± 0.38	1.96 ± 0.03 0.03 ± 0.01
PHEMMAC-50/LIG	0–5 5–168	5.03 ± 0.20 2.01 ± 0.63	1.00 ± 0.04 0.01 ± 0.01

## References

[1] Nagarsenker M.S., Londhe V.Y., Nadkarni G.D. (1990). Preparation and evaluation of liposomal formulations of tropicamide for ocular delivery. Int. J. Pharm..

[2] Bourlais C.L., Acar L., Zia H., Sado P.A., Needham T., Leverge R. (1998). Ophthalmic drug delivery systems–recent advances. Prog. Retin. Eye. Res..

[3] Lang J.C. (1995). Ocular drug delivery conventional ocular formulations. Adv. Drug. Deliv..

[4] Farkouh A., Frigo P., Czejka M. (2016). Systemic side effects of eye drops: A pharmacokinetic perspective. Clin. Ophthalmol..

[5] Mäenpää J., Pelkonen O. (2016). Cardiac safety of ophthalmic timolol. Expert. Opin. Drug. Saf..

[6] Hegde R., Verma A., Ghosh A. (2013). Microemulsion: New Insights into the Ocular Drug Delivery. ISRN Pharmaceutics.

[7] Zhou H.-Y., Hao J.-L., Wang S., Zhang Y., Zhang W.S. (2013). Nanoparticles in the ocular drug delivery. Int. J. Ophthalmol..

[8] Cereda C.M., Brunetto G.B., de Araújo D.R., de Paula E. (2006). Liposomal formulations of prilocaine, lidocaine and mepivacaine prolong analgesic duration. Can. J. Anesth..

[9] Gulsen D., Chauhan A. (2004). Ophthalmic drug delivery through contact lenses. Investig. Ophthalmol. Vis. Sci..

[10] Jung H.J., Chauhan A.A. (2012). Ophthalmic drug delivery by contact lenses. Expert Rev. Ophthalmol..

[11] Kurniawansyah I.S., Sriwidodo S., Mita S.R., Ravi K. (2018). Drug Delivery System by Hydrogel Soft Contact Lens Materials: A Review. J. Pharm. Sci.Res..

[12] Jenkins F.A., White H.E. (2001). Fundamentals of Optics.

[13] Mosmann T. (1983). Rapid colorimetric assay for cellular growth and survival: Application to proliferation and cytotoxicity assays. J. Immunol. Methods.

[14] Malhotra S.L., Minh L.Y., Blanchard L.P. (1983). Thermal Decomposition and Glass Transition Temperature of Poly (methy1 Methacrylate) and Poly (isobuty1 Methacrylate). J. Macromol. Sci. Chem..

[15] Morita S., Ye S., Li G.F., Osawa M. (2004). Effect of glass transition temperature (*Tg*) on the absorption of bisphenol A in poly(acrylate)s thin films. Vib. Spectrosc..

[16] Kang L., Jun H.W., Mani N. (2001). Preparation and characterization of two-phase melt systems of LID. Int. J. Pharmaceut..

[17] Morita S. (2014). Hydrogen-bonds structure in poly (2-hydroxyethyl methacrylate) studied by temperature-dependent infrared spectroscopy, Frontier in Chemistry. Polym. Chem..

[18] Wei Y., Nedley M.P., Bhaduri S.B., Bredzinski X., Boddu S.H.S. (2015). Masking the Bitter Taste of Injectable Lidocaine HCl Formulation for Dental Procedures. AAPS. Pharm. Sci. Tech..

[19] Luo Y., Dalton P.D., Shoichet M.S. (2001). Investigating the Properties of Novel Poly (2-hydroxyethyl methacrylate-co-methyl methacrylate) Hydrogel Hollow Fiber Membranes. Chem. Mater..

[20] Ishiyama C., Higo Y. (2002). Effects of Humidity on Young’s Modulus in Poly (methyl methacrylate). J. Polym. Sci. Part B Polym. Phys..

[21] Bhamra T.S., Tighe B.J. (2017). Mechanical properties of contact lenses: The contribution of measurement techniques and clinical feedback to 50 years of materials development. Cont. Lens Anterior Eye.

[22] Varikooty J., Keir N., Woods C.A., Fonn D. (2010). Measurement of the refractive index of soft contact lenses during wear. Eye Contact Lens.

[23] Weber M.J. (2002). Handbook of Optical Materials.

[24] Vargun E., Sankiri M., Arani B.I., Sankiri N.D., Usanmaz A.I. (2010). Synthesis and Characterization of 2-Hydroxyethyl Methacrylate (HEMA) and Methyl Methacrylate (MMA) Copolymer Used as Biomaterial. J. Macromol. Sci. Part A Pure Appl. Chem..

[25] Lin M., Wang H., Meng S., Zhong W., Li Z., Cai R., Chen Z., Zhou X., Du Q. (2007). Structure and release behavior of PMMA/silica composite drug delivery system. J. Pharm. Sci..

[26] Reinhard C.S., Radomsky M.L., Salzman W.M., Hilton J., Brem H. (1991). Polymeric controlled release of dexamethasone in normal rat brain. J. Control. Rel..

[27] Cypes S.H., Saltzman W.M., Giammelis E.P. (2003). Organosilicate – Polymer drug delivery systems: Controlled release and enhanced mechanical properties. J. Control. Rel..

[28] Frank A., Rath S.K., Venkatraman S.S. (2005). Controlled release from bioerodible polymers: Effect of drug type and polymer composition. J. Control. Rel..

[29] Dilmi A., Bartil T., Yahia N., Benneghmouche Z. (2014). Hydrogels based on 2-hydroxyethylmethacrylate and chitosan: Preparation, swelling behavior, and drug delivery. Int. J. Polym. Mater. Polym. Biomater..

[30] Kim S.K. (1968). Small intestine transit time in the normal small bowel study. Am. J. Roentgenol..

[31] Lee Y.Y., Erdogan A., Rao S.S.C. (2014). How to assess regional and whole gut transit time with wireless motility capsule. J. Neurogastroenterol. Motil..

[32] Khalid S.H., Qadir M.I., Massud A., Ali M., Rasool M.H. (2009). Effect of degree of cross-linking on swelling and drug release behaviour of poly(methyl methacrylate-co-itaconic acid) [P(MMA/IA)] hydrogels for site specific drug delivery. J. Drug. Deliv. Sci. Technol..

[33] Wan L.S.C., Heng P.W.S., Wong L.F. (1993). Relationship Between Swelling and Swelling and Drug Release in a hydrophilic Matrix. Drug. Develop. Ind. Pharm..

[34] Khan S., Ranjha N.M. (2014). Effect of degree of cross-linking on swelling and on drug release of low viscous chitosan/poly (vinyl alcohol) hydrogels. Polym. Bull..

[35] Dinu-PÎrvu C., Ivana S. (2013). A Study of the influence of crosslinking degree on the Physicochemical Properties of Gelatin Microparticles. Cellulose Chem. Technol..

[36] Ferreira L., Vidal M.M., Gil M.H. (2000). Evaluation of poly (2-hydroxyethyl methacrylate) gels as drug delivery systems at different pH values. Int. J. Pharm..

[37] Carbinatto F.M., de Castro A.D., Evangelista R.C., Cury B.S.F. (2014). Insights into the swelling process and drug release mechanisms from cross-linked pectin/high amylose starch matrices. Asian J. Pharmaceut. Sci..

